# Sleep patterns and smartphone use among left-behind children: a latent class analysis and its association with depressive symptoms

**DOI:** 10.3389/fpsyt.2024.1500238

**Published:** 2025-02-03

**Authors:** Xue Han, Cheng-Han Li, Heng Miao, Su Xu, Wen-Jing Yan, Juan Chen

**Affiliations:** ^1^ Prevention and Control Department, Wenzhou Seventh People’s Hospital, Wenzhou, China; ^2^ Emergency Department, Third Affiliated Hospital of Wenzhou Medical University, Wenzhou, China; ^3^ School of Mental Health, Wenzhou Medical University, Wenzhou, China; ^4^ Early Intervention 2, Xi’an Mental Health Center, Xi’an, China; ^5^ Xi’an Institute of Mental Health, Xi’an, China

**Keywords:** sleep patterns, smartphone use, depressive symptoms, latent class analysis, left-behind children

## Abstract

**Background:**

Left-behind children in China face challenges in sleep patterns, technology use, and mental health. This study uses an individual-centered approach to derive behavioral profiles associated with depressive symptoms.

**Methods:**

Data from 131,586 left-behind children aged 8 to 18 years from the Chinese Psychological Health Guard for Children and Adolescents Project were analyzed. Participants were recruited from 569 centers across schools, community institutes, orphanages, and children’s hospitals throughout China. Latent class analysis was conducted using weekday and weekend sleep duration and smartphone use as indicators. Depressive symptoms were assessed using the Center for Epidemiologic Studies Depression Scale (CES-D).

**Results:**

Four distinct classes emerged: Sufficient Sleep Low Users (23.6%), Moderate Sleep Medium Users (25.2%), Limited Sleep High Users (22.1%), and Healthy Sleep Low Users (29.2%). Significant differences in CES-D scores were found between classes (F(3, 131579) = 4929, *p* <.001, η² = 0.101). The Limited Sleep High Users class reported the highest levels of depressive symptoms (M = 11.60, SE = 0.0658), while the Sufficient Sleep Low Users class reported the lowest (M = 3.67, SE = 0.0346). A linear relationship between sleep duration and depressive symptoms was observed. Significant weekday-weekend differences in smartphone use were noted in the unhealthy categories.

**Conclusions:**

This study reveals complex associations between sleep patterns, smartphone use, and depressive symptoms among left-behind children. The identified behavioral profiles provide insights into population heterogeneity and inform targeted intervention strategies. Findings emphasize the importance of addressing both sleep and technology use in mental health initiatives for this vulnerable population.

## 1 Introduction

In China, rapid societal transformations over recent decades, marked by urbanization and migration for work, have led to a growing population of “left-behind” individuals, including children, adolescents, and older adults. This phenomenon has profound implications for mental health across multiple generations. The separation from parents due to migration has been linked to a range of negative mental health outcomes. For instance, Zhang and colleagues found that left-behind older adults experience a higher risk of cognitive decline, highlighting the long-term impact of family separation (1). Furthermore, Li and colleagues demonstrated the association between childhood left-behind experience and reduced quality of life among university freshmen (2). These studies, along with others, underscore the public health need for a deeper understanding of the mental health challenges faced by left-behind populations, particularly children and adolescents. With the increasing infiltration of digital technologies into daily life, how sleep patterns, smartphone usage, and mental health outcomes interplay gains attention by researchers and policymakers alike (3). Of note, this relationship is established among adolescents, whose maturing brains and changing social dynamics increase their susceptibility to potential negative effects associated with sleep disturbances and excessive screen time (4). Identifying behavioral profiles of sleep and smartphone use helps us understand and address the mental health needs of this population. Sophisticated analytics, such as latent class analysis, are being employed to identify subtle patterns of behavior that may be risk or protective factors for psychological well-being within this unique demographic (5). This approach would provide valuable insights not only into the complex relationships between lifestyle and mental health outcomes but also lead to interventions and targeted policies in the promotion of psychological resilience among left-behind children.

With the increasing infiltration of digital technologies into daily life, how sleep patterns, smartphone usage, and mental health outcomes interplay gains attention by researchers and policymakers alike. Of note, this relationship is established among adolescents, whose maturing brains and changing social dynamics increase their susceptibility to potential negative effects associated with sleep disturbances and excessive screen time. Identifying behavioral profiles of sleep and smartphone use helps us understand and address the mental health needs of this population. Sophisticated analytics, such as latent class analysis, are being employed to identify subtle patterns of behavior that may be risk or protective factors for psychological well-being within this unique demographic. This approach would provide valuable insights not only into the complex relationships between lifestyle and mental health outcomes but also lead to interventions and targeted policies in the promotion of psychological resilience among left-behind children.

China has undergone dramatic social and demographic changes in recent decades. From 1980 to 2020, the percentage of urban residents increased from 19.4% to 63.9%, while internal migrants grew from virtually zero to 20.2% of the population. During this same period, the proportion of adults aged 60 years and older rose from 6.9% to 18.7%. These profound societal transformations have created new public health challenges, particularly regarding the mental health impacts on left-behind populations.

Recent evidence highlights concerning mental health effects across different left-behind groups. Studies show that left-behind older adults face elevated risks of cognitive decline and depressive symptoms compared to their non-left-behind counterparts (1). Similarly, left-behind children demonstrate higher rates of depressive symptoms, with meta-analyses indicating a prevalence of 30.7% versus 22.8% in non-left-behind children (6). These mental health disparities appear to persist into young adulthood, as reflected in our current study of university students.

### 1.1 Previous research on sleep, smartphone use, and mental health

Much attention has been paid to problems with mental health, in particular depression symptoms, among left-behind children in China. A meta-analysis reported that the pooled prevalence of depressive symptoms in left-behind children was 30.7% compared to 22.8% in non-left-behind children, with left-behind children being significantly more likely to suffer from depressive symptoms (6). This has been explained by various reasons, one among them has been less parental supervision, emotional deprivation, and socio-economic disadvantages.

Concurrently, it has also been focused on the research regarding the impact of sleep patterns and smartphone usage on the mental health of adolescents. More recently, a longitudinal study showed that youth who had increased electronic communication reported more symptoms of depression over time; each additional hour significantly predicted a 0.76-point increase in depressive symptoms (7). Similarly, the use of smartphones at night has been observed to be linked to the shortening of sleep duration among young individuals, as well as higher levels of depressive symptoms (8).

The relations between sleep and mental health have also been well documented in the adolescent population. A systematic review reported that sleep duration was negatively associated with depressive symptoms; adolescents who reported sub-optimal sleep were 1.5 to 2 times more likely to experience elevated depressive symptoms (9). Furthermore, a higher likelihood of depression was associated with both inadequate and excessive sleep durations, thereby indicating a U-shaped function between sleep time and depressive symptoms (10).

To date, few studies have started to investigate the combined effects of sleep and smartphone use on adolescent mental health. For instance, studies showed that problematic smartphone use was related to sleep disturbances and depressive symptoms; the relationship was partially mediated by sleep quality (11). However, most of the previous studies have adopted variable-centered approaches, which may mask important subgroup differences in these behavioral patterns.

The application of person-centered approaches, such as latent class analysis, in understanding the patterns of sleep and smartphone use is relatively recent. One study employed latent class analysis to identify distinct patterns of sleep problems among Chinese adolescents and investigated their associations with internalizing and externalizing problems (12). Similarly, one study utilized latent class analysis to examine gender-specific differences in problematic smartphone and internet use among Korean middle school students, revealing that males are more prone to internet-related issues, while females demonstrate a higher propensity for smartphone-related problems (13).

Few studies have examined the combined effects of sleep patterns and smartphone use on depressive symptoms among left-behind children in China. In addition, important weekday and weekend behaviors, which might provide a more detailed understanding of the adolescent lifestyle, have not been thoroughly addressed in previous works.

### 1.2 Gaps in current knowledge

Few studies have addressed two key issues, however, in this rapidly expanding body of research on sleep patterns, smartphone use, and mental health among adolescents: the integration of the sleep patterns and smartphone use within one comprehensive behavioral profile and taking place within the context of being left behind in China. This is necessary if the complex interaction between these lifestyle factors is to be captured and their impact on mental health outcomes optimized.

Second, most current research uses variable-centered approaches that may mask important subgroup differences and not capture the heterogeneity of behavioral patterns in this population. Much less attention has been paid, for instance, in the application of person-centered approaches, such as latent class analysis, toward joint exploration of sleep and smartphone use patterns, especially in the context of left-behind children (5).

Third, very few of the earlier studies have distinguished between weekday and weekend behaviors. This differentiation is necessary to capture the full spectrum of adolescent lifestyles, as patterns between school days and free days may vary importantly and then impact differently on mental health outcomes.

Fourth, while the vulnerability of these children to mental health issues is established, there are very few studies relating specific behavioral patterns to sleep and smartphone usage, which could respectively add to or subtract from depressive symptoms among such children. Of note, the uniqueness of left-behind children warrants this knowledge to design targeted interventions and policies (14).

In addition, the distinction between weekday and weekend behaviors is particularly relevant for left-behind children due to their unique circumstances. During weekdays, these children are typically under school supervision, following structured routines that regulate both sleep patterns and technology use. However, weekends present a different scenario where the absence of parental oversight may be more acutely felt. This weekday-weekend comparison becomes crucial as it may reveal behavioral patterns that are specifically linked to their left-behind status.

### 1.3 The present study

To address these gaps, the present study aims to identify distinct classes of sleep and smartphone use patterns among left-behind children in China, considering both weekday and weekend behaviors. We also try to examine the association between these identified classes and depressive symptoms, as measured by the Center for Epidemiologic Studies Depression Scale (CES-D). What is more, we hope our study can provide a more nuanced understanding of the complex relationships among sleep patterns, smartphone use, and depressive symptoms in left-behind children in China. Such knowledge may be used to inform the development and implementation of targeted interventions and policies for the promotion of mental health in this vulnerable population.

## 2 Method

### 2.1 Participants and procedures

This study utilized data from the Chinese Psychological Health Guard for Children and Adolescents Project (15), which is a large-scale, multi-center cohort study set up to evaluate the impact of primary psychological health care on depression and suicidal ideation in underserved children and adolescents. Through a “2 + 2” primary psychological health care system workflow, information was gathered in the left-behind cohort from October 2022 (baseline) to May 2023 (half-year follow-up): two rounds of psychological screening, followed by two rounds of early psychological intervention as deemed necessary.

From an original sample of 179,877 left-behind children, 131,586 who completed the CES-D (Centers for Epidemiologic Studies Depression Scale) aged between 8 and 18 years are included in the final sample. Data were collected at 569 centers throughout school, community-based institutes, orphanages, and children’s hospitals. All the procedures of the study were confirmed by the Institutional Review Board of Nanchong Psychosomatic Hospital (No. NCPP 2022002), and informed consent by parents or legal guardians was obtained.

### 2.2 Measures

The primary outcome measure was the Center for Epidemiologic Studies Depression Scale (CES-D)—20 items well validated to screen depressive symptoms in nonclinical populations (16). CES-D is a 20-item scale scored using a 4-point Likert scale, which measures how frequently depressive experiences are over a week. Response categories vary from 0 points for “rarely or none of the time (<1 day)” to 3 points for “most or all of the time (5-7 days)”. Total scores ranged from 0 to 60, with higher scores indicating a higher level of depression symptomatology. A cut-off score of ≥16 was used to screen people with clinically significant depressive symptoms. The psychometric properties of the CES-D are strong when used with Chinese adolescents (16). In an effort to minimize the errors during data entry and to standardize the administration, CES-D was administered using a custom-made Smartphone application that was compatible with multiple operating systems such as iOS and Android. The internal consistency reliability (Cronbach’s α) in the present study was 0.94.

Demographic data, including gender, age, place of residence (urban, rural) whether the child was an only child in the family was abstracted from digital records in resident/school registration systems, and confirmed by participants. The measures on sleep patterns and smartphone use are presented in this paper. Four self-reported indicators were included for identification of distinct subgroups in these variables: (1) weekday sleep duration, average hours of sleep on school nights; (2) weekend sleep duration, average hours of sleep on weekend nights; (3) weekday smartphone use, average daily hours of smartphone use on school days; and (4) weekend smartphone use, average daily hours of smartphone use on weekend days. This dual measurement approach, taking both school days and free days as measurements, enabled the identification of potential discrepancies important for understanding the behavioral patterns of adolescents.

Simple characteristics for this participant can be found in **Table 1**.

**Table 1:** Sample characteristics of the left-behind children (*N*= 131,586).

Variable	Category	Unweighted n	Weighted %
Gender			
	Female	64593	49.09
	Male	66990	50.91
Living Area			
	Urban	50106	38.08
	Rural	81477	61.92
Only Child			
	Yes	24009	18.25
	No	107574	81.75
Weekday Sleep			
	Less than 5 hours	3591	2.73
	5 - 6 hours	28753	21.85
	7 - 8 hours	65624	49.87
	9 - 10 hours	30268	23.00
	More than 10 hours	3347	2.54
Weekend Sleep			
	Less than 5 hours	3929	2.99
	5 - 6 hours	10275	7.81
	7 - 8 hours	47243	35.90
	9 - 10 hours	55213	41.96
	More than 10 hours	14923	11.34
Weekday Smartphone Use			
	Never used	57632	43.80
	Less than 1 hour	46429	35.28
	1 - 2 hours	17918	13.62
	2 - 3 hours	5697	4.33
	3 - 4 hours	1899	1.44
	More than 4 hours	2008	1.53
Weekend Smartphone Use			
	Never used	15400	11.70
	Less than 1 hour	28349	21.54
	1 - 2 hours	33331	25.33
	2 - 3 hours	22468	17.08
	3 - 4 hours	12779	9.71
	More than 4 hours	19256	14.63

### 2.3 Data analysis

We applied LCA through R (version 4.1.0) with the package poLCA (17) to identify discrete profiles of sleep patterns and smartphone use between adolescents. We included 4 continuous indicator variables: weekday and weekend sleep duration, and weekday and weekend smartphone use—measured in hours. Covariates included in the model are age, gender, living area, and only child status. Missing values were treated by a complete case analysis. While this may come at the cost of some bias if data are not actually missing completely at random, it is a very simple way to handle missing values in an LCA framework.

Successive-class models were estimated, and comparisons made using Bayesian Information Criterion (BIC), Sample-Size Adjusted BIC (aBIC), Consistent Akaike Information Criterion (CAIC), Approximate Weight of Evidence (AWE), bootstrap likelihood ratio test (BLRT) and Vuong-Lo-Mendell-Rubin Likelihood Ratio Test (VLMR-LRT). Finally, relative entropy was further scrutinized as an additional diagnostic criterion.

According to the clear features of each defined class, we gave it a descriptive name. These names had been chosen to reflect the most prominent characteristics of sleep patterns and smartphone use that distinguished one class from others. We subsequently conducted a one-way analysis of variance (ANOVA) with the CES-D scores as the dependent variable and the latent class membership as the independent variable to test for potential between-class differences in depressive symptoms. These results helped establish significant differences in the severity of depressive symptoms between classes. *Post hoc* tests were also carried out, using Tukey’s HSD, for instances in which the overall ANOVA was significant, to determine which differences between classes were specific.

## 3 Result

### 3.1 Latent classes for sleep patterns and smartphone use

**Figure 1:**
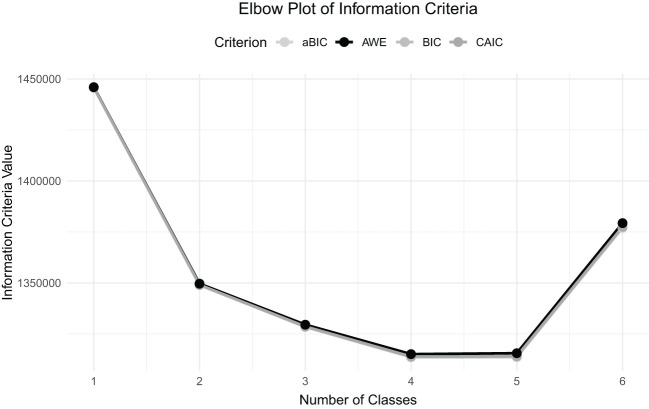
Elbow plot of the information criteria for latent class models with 1 to 6 classes. The plot represents four measures: the Adjusted Bayesian Information Criterion (aBIC), the Approximate Weight of Evidence (AWE), the Bayesian Information Criterion (BIC), and the Consistent Akaike Information Criterion (CAIC) with corresponding number of classes. The elbow point is that point where further increasing the classes beyond that number doesn’t add much to the improvement in model fit, which in this case falls over the 4-class solution—thereby supporting this model.

We considered multiple fit criteria to compare estimated latent class models with 1 to 6 classes (see **Table 2**). The Bayesian Information Criterion (BIC) is one of the most reliable indications of model fit in the current model comparison literature (18), and it pointed out that the best fit solution was a 4-class solution (BIC = 1,315,301). Again, the sample-size adjusted BIC (aBIC) and Consistent Akaike Information Criterion (CAIC) both pointed to the 4-class model as the best fitting to the data (aBIC = 1,315,024; CAIC = 1,315,388). **Figure 1** provides a visual representation of these information criteria.

**Table 2:** Fit statistics for latent class analysis models with 1 to 6 classes.

Class	*Npar*	*LL*	*BIC*	*aBIC*	*CAIC*	*AWE*	*RE*	*BLRT.p*	*VLMR-LRT.p*
**Class-1**	18	-722752	1445715	1445658	1445733	1445981	NA	NA	NA
**Class-2**	41	-674281	1349046	1348916	1349087	1349652	0.729	<.001	<.001
**Class-3**	64	-663926	1328606	1328402	1328670	1329552	0.737	<.001	<.001
**Class-4**	87	-657138	1315301	1315024	1315388	1316587	0.770	<.001	<.001
**Class-5**	110	-658200	1317697	1317347	1317807	1319323	NA	1	1
**Class-6**	133	-656420	1314407	1313984	1314540	1316374	NA	<.001	<.001

The bold text in the column headers represents various statistical metrics used in latent class analysis: Npar, Number of parameters in the model; LL, Log-Likelihood; BIC, Bayesian Information Criterion; aBIC, Adjusted Bayesian Information Criterion; CAIC, Consistent Akaike Information Criterion; AWE, Approximate Weight of Evidence; RE, Relative Entropy; BLRT.p, Bootstrap Likelihood Ratio Test p-value; VLMR-LRT.p, Vuong-Lo-Mendell-Rubin Likelihood Ratio Test p-value. NA, Not Applicable.

The results of the Bootstrap Likelihood Ratio Test (BLRT) and Vuong-Lo-Mendell-Rubin Likelihood Ratio Test (VLMR-LRT) revealed that the 4-class solution significantly improved the model fit compared to the 3-class solution (both *p* <.001). Also, with respect to the 4-class model and very much in line with this, the addition of a 5th class did not significantly improve the solution, according to the non-significant BLRT and VLMR-LRT test results (*p* = 1 for the 5-class solution). In line with these findings, the 6-class solution was significant according to these tests (*p* <.001); however, by the rest of the criteria, preference remained with the 4-class model.

The relative entropy values are again the indicators of classification quality and were observed for the 2-, 3-, and 4-class solutions. The 4-class model presented higher entropy (*RE* = 0.77) than the 2-class (*RE* = 0.729) and 3-class (*RE* = 0.737) models, respectively, and had better classification quality overall. Based on these statistical criteria and the interpretability of the classes, the final model was based on the 4-class solution. This decision was drawn on the ground of the smallest BIC, aBIC, and CAIC values, with substantial improvement over the three-class model, and highest in relative entropy among the tested solutions.

### 3.2 Class characteristics

Class 1: Adequate Sleep Low Users (23.6%). This class shows long sleep duration but low smartphone use. Sleep duration on weekdays is predominately 9 hours and more (97.15%) and on weekends, it is also predominantly 9 hours and more (90%). Smartphone use is very low, with 89.36% spending less than 1 hour or even never using it on weekdays; this proportion remains high on weekends at 60.03%. This group tends to have adequate time for sleep and minimal reliance on their smartphone.

Class 2: Average Sleep Average Users (25.2%). This class indicates an average period of sleeping and average users of smartphones. Regarding the duration of sleep on weekdays, it is majorly 7-8 hours (89.4%), while it slightly increases over the weekends but still remains at average values. The use of smartphones tends to be low during weekdays while high during the weekend. 67.2% use the phone for less than 1 hour or never use it during the weekdays, and 94.49% use it for 2 hours or more on weekends. This pattern indicates a considerable difference in sleep and phone use between weekdays and weekends.

Class 3: Limited Sleep High Users (22.1%). Short sleep duration combined with high smartphone usage are the major characteristics of this category. It is observed that 99.17% sleep no more than 6 hours on weekdays and 31.6% still sleep for a duration less than 6 hours on weekends. Smartphone use duration is seen to be relatively long, especially on weekends, in 65.84% who use it for 2 hours or more. This shows an inverse possible correlation between the durations of sleep and smartphone use.

Class 4: Healthy Sleep Low Users (29.2%). In this category, students have healthy sleep patterns and low smartphone use. 94.31% sleep between 7 and 8 hours during the weekdays, and 92.25% sleep between 7 and 10 hours over the weekend. The usage duration of smartphones generally lasts for a very short duration: 91.95% use it for less than one hour or not at all on weekdays, while over the weekend, 100% use it for not more than 2 hours. This shows a healthy pattern of life regarding the adequate time spent sleeping and on smartphone usage.

**Figure 2:**
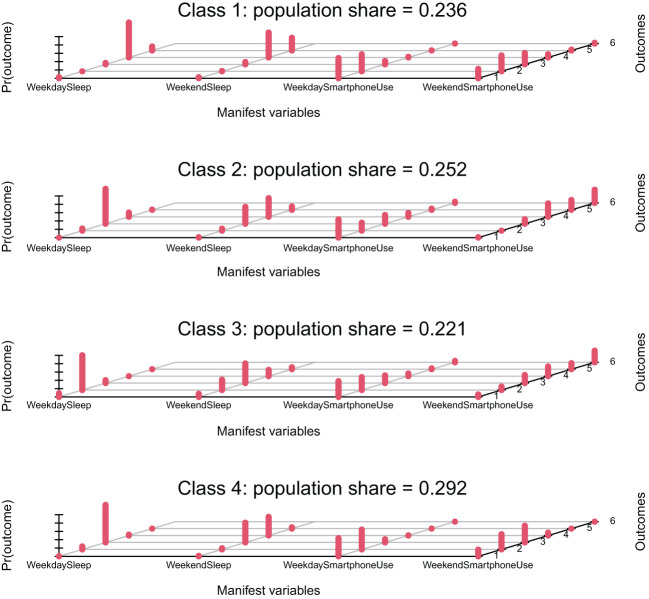
Latent classes of sleep duration and smartphone use patterns among adolescents. The x-axis shows the four manifest variables: Weekday Sleep, Weekend Sleep, Weekday Smartphone Use, and Weekend Smartphone Use. The y-axis represents the probability of each response category within these variables.

Our latent class analysis revealed four distinct patterns of sleep and smartphone use among adolescents, each with potential implications for behavioral science and mental health (see **Figure 2**). The “Sufficient Sleep Low Users” and “Healthy Sleep Low Users” classes both exhibited positive behaviors, characterized by adequate sleep duration and limited smartphone use. These classes could be considered healthier sleep patterns for adolescents, potential protective factors against mental health problems, and examples for healthful lifestyle promotion.

The “Limited Sleep High Users” class showed an alarming pattern of limited sleep hours but high use of smartphones, especially on weekend nights. This group of individuals might be at risk for a more intensified mental health problem state because of the possible negative consequences of cognitive function and emotional regulation due to sleep deprivation or excessive technology use. The correlation between sleep duration and the use of smartphones in this group is negative, indicating that one might trade off against the other; this corroborates other recent studies on the negative impact of nighttime technology use with respect to sleep quality and duration.

For the class “Moderate Sleep Medium Users,” there were interesting weekday-weekend differences in their sleep and smartphone usage patterns. This group exhibited significantly higher use of smartphones on weekends yet moderate durations of sleep. This incongruence in sleep and technology use schedules may compromise circadian rhythms and, in turn, quality of sleep and mental health.

### 3.3 The differences in CES-D scores across different class membership

Thus, to test the prediction from the latent class membership to the depressive symptom variable, a one-way ANOVA test was made, using CES-D scores as the dependent variable and the predicted latent class membership as an independent variable. The result showed statistically significant differences of the CES-D score among the four latent classes, *F*(3, 131579) = 4929, *p* <.001, *η²* = 0.101.

**Figure 3:**
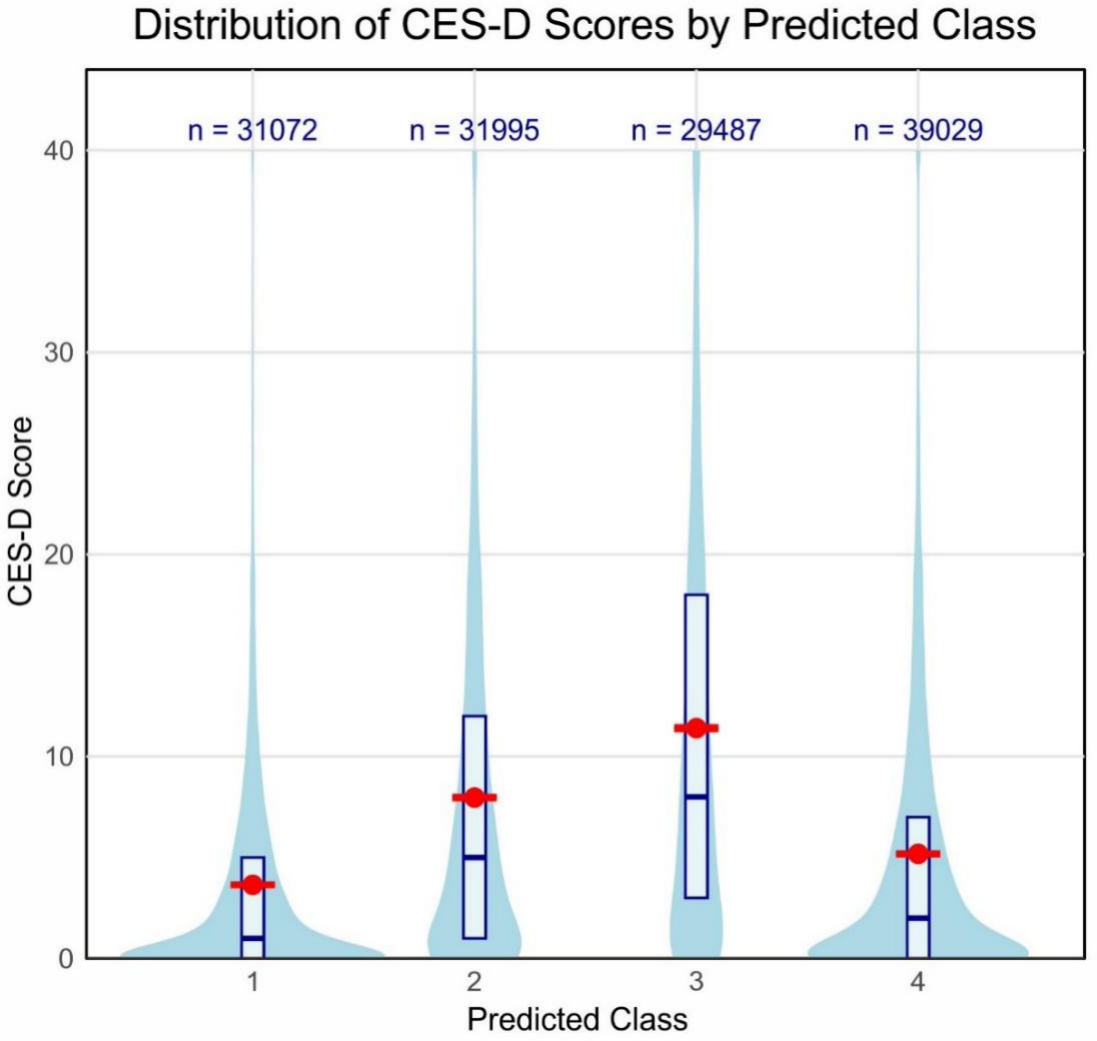
Distribution of CES-D scores by predicted latent class. The violin plot shows the distribution of CES-D scores for each predicted class. Red dots and error bars represent mean scores and standard errors, respectively. Box plots within each violin indicate median and interquartile ranges. Sample sizes for each class are shown at the top of the plot.

Descriptive statistics indicated that Class 3, Limited Sleep High Users, had the highest mean CES-D score (*M* = 11.60, *SE* = 0.0658), followed by Class 2, Moderate Sleep Medium Users (*M* = 8.04, *SE* = 0.0522); Class 4, Healthy Sleep Low Users (*M* = 5.22, *SE* = 0.0385); and Class 1, Sufficient Sleep Low Users (*M* = 3.67, *SE* = 0.0346). **Figure 3** below shows the distribution of CES-D scores across the four latent classes. This diagram presents good illustration for model-based differences.

*Post hoc* comparisons, using Tukey’s HSD test, indicated statistically significant differences for all pairs of classes (*p* < 0.001 in each case). The highest mean difference was observed between Class 3 and Class 1 and equal to 7.91 points, with 95% CI [7.73, 8.09], while the lowest significant difference was between Class 4 and Class 1; it equaled 1.55, 95% CI [1.38, 1.72]. Eta-squared (η²) is 0.101, indicating that around 10.1% of the variance in CES-D scores can be explained by class membership; this should be moderate to large in terms of effect size.

These findings establish the clear association between the patterns of sleep and smartphone use and the depressive symptoms of adolescents. This was evident with the class characterized by low sleep and high smartphone use (Class 3), where depressive symptoms were considerably high compared to all other classes, especially those characterized by sufficient sleep and low smartphone use (Class 1). A look at **Figure 3** easily tells this fact, as the violin plot for Class 3 has a larger majority of scores in the upper ends of the CES-D scale. This underpins the potential mental health implications of sleep deprivation and excessive smartphone use among the adolescent population.

## 4 Discussion

The current study differentiated the latent classes of sleep patterns and smartphone use of a left-behind child sample in China and their associations with depressive symptoms. Using the LCA approach, we identified four distinguishable behavioral classes: Sufficient Sleep Low Users (23.6%), Moderate Sleep Medium Users (25.2%), Limited Sleep High Users (22.1%), and Healthy Sleep Low Users (29.2%). These classes demonstrated unique proportions of weekday and weekend sleep hours, and patterns of smartphone use that reflected the diversity in adolescent behavior. Another important outcome here was that these behavior profiles showed high correlation with depressive symptoms quantified by CES-D. The Low Sleep High Users class manifested much higher levels of depressive symptoms compared to those in the other remaining classes, in which those adolescents had adequate sleep and low use of smartphones.

### 4.1 Sleep patterns, smartphone use, and their combined impact on depressive symptoms

Our latent class analysis discovered four different profiles in terms of sleep and smartphone use among left-behind children in China, and these were linked with varying levels of depressive symptoms. This multidimensional approach provides a nuanced understanding of how these behaviors interact and jointly influence mental health outcomes. Those in the “Sufficient Sleep Low Users” class, reporting long sleep duration (≥9 hours) and minimal smartphone use, showed the lowest level of depressive symptoms (M = 3.67, SE = 0.0346). This is congruent with recent work, which indicates that longer sleep is associated with better mental health among youth (19). However, our findings further enriched the understanding by suggesting that sleep durations beyond the generally recommended 8-9 hours might bring additional mental health benefits to left-behind children.

On the other hand, the highly significant symptoms of depression were among the “Limited Sleep High Users” class (*M* = 11.60, *SE* = 0.0658). The study found a consistent relationship between insufficient sleep, frequent smartphone use and negative effects on mental health, with evidence suggesting a strong link between problematic smartphone use and adolescent depression (20–22). Our study adds to this by showing that sleep deprivation and excess smartphone use multiply to add to depressiveness, particularly in the context of left-behind children.

We have observed a very clear linear relationship in the sleep duration and mental health outcomes across all classes, where longer sleep times were consistently linked to lower depressive symptoms. This suggests a challenge to the U-shaped relationship of sleep duration with depression, which is often reported in previous studies (23, 24). This divergence is possibly due to the special circumstance of being left behind, which may have either reinforced having more hours of sleep or reduced external pressure.

### 4.2 The interplay between smartphone use and sleep patterns

Although within the “high use” category, the weekday use was mostly below 2 hours, on average, our results suggest an unexpected pattern of relatively low overall smartphone use for the adolescents in our sample, particularly on weekdays. This contrasts with the prevalent narrative of excessive smartphone use among teenagers reported in studies (22, 25). Yet, moderate levels of such combinations as less sleep and more smartphone use in a single class still had positive associations with depressive symptoms, indicating that the harmful effects could interact with limited sleep. This is borne out by recent research work (8), who found that nighttime smartphone use was associated with both reduced sleep quality and increased depressive symptoms in adolescents.

The weekday-weekend gap in such smartphone use, Class 2, can be seen to echo findings by a previous study (26), who found that adolescent sleep and technology use differed greatly between school and free days. Our study extends this general understanding to the context of left-behind children in China, suggesting that the factor of missing parental supervision enhances these temporal variations in behavior. Additionally, the “Limited Sleep High Users” class showed an inverse relationship between sleep duration and smartphone use, hence supporting the displacement hypothesis, which posits that time spent on smartphones directly competes with sleep time (8). However, we found that this association might be stronger in certain subgroups of adolescents, meaning that person-centered approaches are needed to reveal such complex behavioral patterns.

### 4.3 Behavioral patterns and psychological resilience of left-behind children

As a result of our findings, current views on the vulnerability of “left-behind children” are challenged; for example, a high proportion of our sample demonstrated good behavioral patterns. More specifically, “Sufficient Sleep Low Users” (23.6%)and “Healthy Sleep Low Users” (29.2%) classes indicated adaptive sleep and smartphone use behaviors, which related to lower levels of depressive symptoms. This may echo recent research (27), who reported that children left behind in China often learn to adapt to the situation of parental absence. Our work further explicates this by pointing out particular behavior patterns that may lead to psychological resilience. Inasmuch as a large majority of these left-behind children exhibit healthy sleep habits and moderate use of technology, it may be a hint of the possibility of positive adjustment.

Of note, heterogeneity in our sample was reflected by the class of “Limited Sleep High Users” at 22.1% who were significantly more depressed in the context of insufficient sleep and high smartphone usage. Higher risk subgroups of left-behind children with mental health issues have been identified, consistent with previous research (28, 29). Our results suggest that sleep patterns and technology use may be key factors differentiating between resilient and vulnerable subgroups within this population.

The reasons for observed resilience among some left-behind children are multiple. First, the absence of parental surveillance might paradoxically lead to more consistent sleep schedules and less access to smartphones, especially in rural areas with low technological infrastructure. This hypothesis is supported by recent work (30) showing that for some left-behind children, reduced adult oversight led to an increase in self-regulation.

Alternative caregivers, such as grandparents or other relatives, might contribute to keeping patterns of healthful behavior intact. In fact, Li and colleagues note that quality relationships with alternative caregivers might be a key reason for relative resilience of left-behind children against many deleterious mental health outcomes. Our results here suggest that these caregiving relationships may also relate to daily patterns of sleep and technology use.

Resilience, as found in our study, carries an important message for left-behind children’s interventions. The generalized vulnerability model with left-behind children should be abandoned in favor of a more nuanced one, identifying and supporting those at highest risk while leveraging and reinforcing adaptive behaviors in more resilient groups.

### 4.4 The impact of weekday-weekend behavioral differences

The class of “Moderate Sleep Medium Users” that showed an increasing pattern in weekend smartphone use could belong to groups particularly prone to such behavioral inconsistencies. This pattern is also somewhat resonant with recent work (31), which demonstrated that higher discrepancies in sleep timing and duration between weekdays and weekends predicted more symptoms of depression and anxiety in adolescence. As such, our results would seem to indicate that such mechanisms are also present in left-behind children, albeit perhaps less straightforward due to decreased parental supervision. The influence of school systems on managing adolescents’ actions is seen to dominate in our trial as well, particularly from the data of lesser quantity of weekday smartphone usage for each of the grades, which is consistent with a study on urban Chinese children found that prolonged time spent on homework and mobile phone use was related to shorter sleep duration and later bedtimes. This indicates that school-related activities and routines directly influence sleep patterns (32). Our results indicate that the protective role of these structures may be nullified for left-behind children over weekends when children generally do not stay at schools and receive relatively less supervision or planned activities.

These weekday-weekend behavioral differences raise questions about their long-term impact on children? A longitudinal research (33) suggested that consistent routines are one crucial element for healthy psychological development during adolescence. The irregular patterns observed in our study may disrupt the attainment of stable circadian rhythms and good habits, thus setting these young adults up for increased vulnerability to mental health issues over time. In contrast, the Sufficient Sleep Low Users (Healthy Class 1) show very regular sleep and smartphone use on weekdays and weekends.

These findings have implications for such interventions. Programs should provide structure and support during weekdays, weekends, and holidays. This may mean community-based interventions to offer structured activities, promote consistent sleep schedules, and balanced technology use throughout the week. In addition, educating alternative caregivers to maintain these routines may ameliorate the impact of weekday–weekend behavioral discrepancies.

### 4.5 Limitations and implications

There are a few limitations in our study. First, because of the nature of the cross-sectional study, it could not infer causality for the relationships between sleep patterns, smartphone use, and depressive symptoms (34). Second, self-reported measures were used, so recall bias and social desirability effects might occur (35). Third, although the sample size was large, it cannot be said to represent all left-behind children in China since there might be regional variations. Fourth, our assessment of sleep patterns was limited to sleep duration on weekdays and weekends. While this provides a valuable initial understanding, it does not capture other crucial aspects of sleep, such as sleep quality, onset latency, or sleep-wake regularity.

Our identification of distinct behavioral classes suggests the need for targeted interventions. For the Short Sleep-Heavy Tech Users class, interventions should focus on establishing strict bedtime routines and implementing technology-free periods before sleep, possibly through school-based programs that can compensate for absent parental oversight. The Standard Sleep-Weekend Tech Users class would benefit from weekend activity programs that provide structured alternatives to excessive smartphone use. For the Long Sleep-Minimal Tech Users and Optimal Sleep-Consistent Tech Users classes, interventions should focus on maintaining their positive behaviors through peer support networks and reward systems.

## 5 Conclusion

This study found the patterns of sleep and smartphone use in left-behind children in China, relating these patterns with depressive symptoms. Longer sleep and less smartphone use correlate with better mental health. Contrary to common beliefs about left-behind children’s vulnerability, many participants showed positive behavioral patterns. However, the association of the class with limited sleep, high smartphone use, and increased depressive symptoms underscores potential risks. This is an important insight to develop an understanding of the complicated interaction of daily behaviors on mental health in this population. Future research and interventions should focus on promoting healthy sleep habits and responsible technology use in support of well-being for China’s left-behind children in its changing social landscape.

## Data Availability

The original contributions presented in the study are included in the article/supplementary material. Further inquiries can be directed to the corresponding author.
